# Artemisinin ameliorates cognitive decline by inhibiting hippocampal neuronal ferroptosis via Nrf2 activation in T2DM mice

**DOI:** 10.1186/s10020-024-00797-9

**Published:** 2024-03-07

**Authors:** Bo Wang, Sheng Zhu, Miao Guo, Run-Dong Ma, Ya-Ling Tang, Ya-Xiong Nie, Hong-Feng Gu

**Affiliations:** 1 Institute of Anesthesiology, The First Affiliated Hospital, Hengyang Medical School, University of South China, Hengyang, 421001 Hunan China; 2 Department of Nuclear Medicine, Affiliated Hospital of Xiangnan University, No. 25 Renmin West Road, Beihu District, Chenzhou, 423001 Hunan China; 3 Department of Physiology and Institute of Neuroscience, Key Laboratory of Hunan Province for Major Brain Diseases, Hengyang Medical School, University of South China, Hengyang, 421001 Hunan China

**Keywords:** Diabetic cognitive deficit, Ferroptosis, Hippocampus, Artemisinin, Nrf2

## Abstract

**Background:**

Neuronal ferroptosis plays a critical role in the pathogenesis of cognitive deficits. The present study explored whether artemisinin protected type 2 diabetes mellitus (T2DM) mice from cognitive impairments by attenuating neuronal ferroptosis in the hippocampal CA1 region.

**Methods:**

STZ-induced T2DM mice were treated with artemisinin (40 mg/kg, i.p.), or cotreated with artemisinin and Nrf2 inhibitor MEL385 or ferroptosis inducer erastin for 4 weeks. Cognitive performance was determined by the Morris water maze and Y maze tests. Hippocampal ROS, MDA, GSH, and Fe^2+^ contents were detected by assay kits. Nrf2, p-Nrf2, HO-1, and GPX4 proteins in hippocampal CA1 were assessed by Western blotting. Hippocampal neuron injury and mitochondrial morphology were observed using H&E staining and a transmission electron microscope, respectively.

**Results:**

Artemisinin reversed diabetic cognitive impairments, decreased the concentrations of ROS, MDA and Fe^2+^, and increased the levels of p-Nr2, HO-1, GPX4 and GSH. Moreover, artemisinin alleviated neuronal loss and ferroptosis in the hippocampal CA1 region. However, these neuroprotective effects of artemisinin were abolished by Nrf2 inhibitor ML385 and ferroptosis inducer erastin.

**Conclusion:**

Artemisinin effectively ameliorates neuropathological changes and learning and memory decline in T2DM mice; the underlying mechanism involves the activation of Nrf2 to inhibit neuronal ferroptosis in the hippocampus.

**Graphical Abstract:**

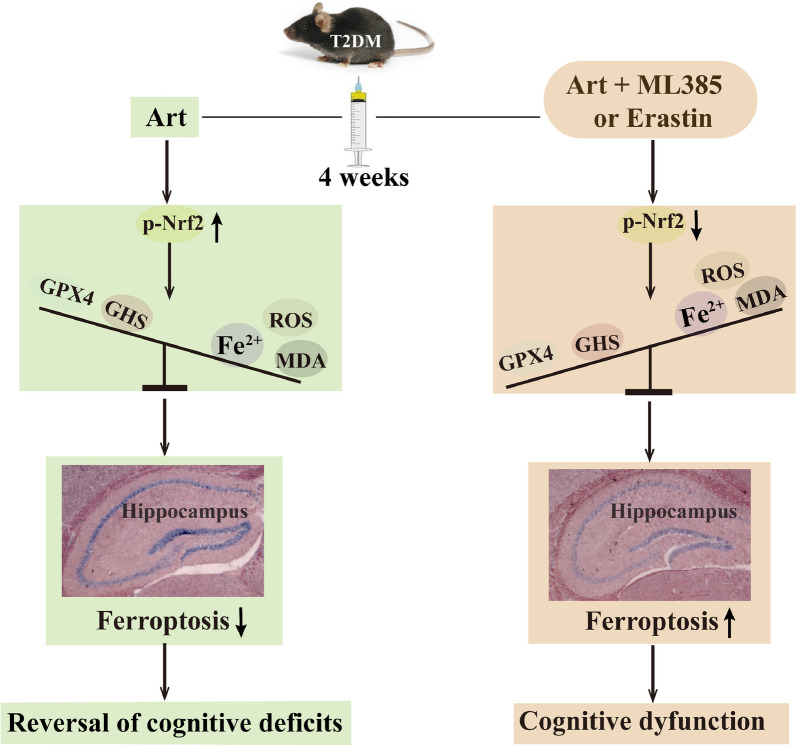

## Introduction

Diabetes mellitus (DM) is one of the most common metabolic diseases characterized by hyperglycemia resulting from insulin deficiency or resistance. Diabetes often causes various complications, such as kidney disease, retinopathy, cardiovascular disease, peripheral neuropathy, and cognitive dysfunction. Among these complications, DM is closely associated with learning and memory deficits (Liu et al. 2020; Luo et al. 2022; McCrimmon et al. 2012). Epidemiological data and clinical studies have confirmed that up to 50% of patients with type 2 diabetes mellitus (T2DM) present cognitive dysfunction (Chatterjee et al. 2016). The pathogenesis of diabetic cognitive dysfunction is complicated, including dysglycemia, oxidative stress, inflammatory response, and insulin resistance (Hao et al. 2021). Due to the underlying mechanism of diabetes-associated cognitive dysfunction remains unclear, there is no effective drug treatment for this major complication (Luo et al. 2022; Wolf et al. 2021; Zhang et al. 2021). As such, a better understanding of its mechanisms is of urgent importance for developing strategies to prevent and treat diabetic cognitive impairments.

Ferroptosis is a novel type of programmed cell death triggered by iron-dependent lipid peroxidation (Wang 2023; Zou et al. 2020). Morphologically, ferroptosis is featured by increased mitochondrial density, disrupted mitochondrial cristae, and mitochondrial shrinkage (Yang et al. 2021). Its potential mechanism is mainly associated with antioxidant system dysregulation, such as glutathione peroxidase 4 (GPX4) inactivation, glutathione (GSH) depletion, and iron overload (Wang 2023; Zou et al. 2020). The dysregulated antioxidant systems together with imbalanced iron homeostasis trigger lipid peroxidation, which then results in the accumulation of reactive oxygen species (ROS) and eventually causes cell death. Substantial evidence has revealed that ferroptosis plays a critical role in neurodegenerative disorders such as Alzheimer’s disease and Parkinson’s disease (Bao et al. 2021; Wang et al. 2022). In addition, recent studies have revealed that ferroptosis is also implicated in the pathogenesis of T2DM and its complications (Guo et al. 2023; Li et al. 2020; Xie et al. 2023). Tang et al. found that ferroptosis contributes to the initiation and development of diabetic cognitive dysfunctions, which can be rescued by ferroptosis inhibitors (Tang et al. 2022). Although several ferroptosis inhibitors have been identified, a majority of them are iron chelators or small antioxidants (Yang et al. 2022). The use of these inhibitors inevitably results in serious side effects, including anemia and metabolic disorders (Xi et al. 2022). Hence, it is of great importance to develop novel ferroptosis inhibitors with few side effects.

Artemisinin (Art), a natural compound derived from the plant *Artemisia annua*, possesses potent anti-malaria, anti-inflammatory, and antioxidant properties (Cao et al. 2020; Kong and Tan 2015). This medicine has no observable side effects and can permeate the blood–brain barrier (Jiang et al. 2020; Shi et al. 2013; Zhao et al. 2020). Recently, Art and its derivatives have been shown to have neuroprotective and pro-cognitive effects by protecting neurons from oxidative injury (Zhao et al. 2020). Albasher G et al. also demonstrated that *Artemisia judaica* extract attenuates the neuronal impairments associated with diabetes (Albasher et al. 2020). Furthermore, Shao et al. revealed that dihydroartemisinin (a derivative of artemisinin) exerts its antiepileptic effects by inhibiting hippocampal neuron ferroptosis (Shao et al. 2022). Collectively, these results suggest that Art may attenuate diabetic cognitive dysfunction by inhibiting ferroptosis in hippocampal neurons.

This study aimed to clarify whether Art mitigates diabetic cognitive decline through Nrf2-mediated anti-ferroptosis. We first assessed the influences of Art on cognitive performance, Nrf2 and GPX4 expression, and hippocampal neuronal ferroptosis in T2DM mice. We then explored whether the neuroprotective and pro-cognitive effects of Art were reversed by the Nrf2 inhibitor ML385 or the ferroptosis inducer erastin. We demonstrated that Art dramatically improved cognitive performance, activated the Nrf2/GPX4 pathway, and alleviated neuronal ferroptosis in the hippocampus of T2DM mice. However, these beneficial effects of Art were abolished by ML385 or erastin. Together, the present study reveals that Art attenuates diabetic cognitive impairments by activating Nrf2 to inhibit neuronal ferroptosis.

## Materials and methods

### Materials

Art (purity ≥ 98%), erastin, and ML385 were purchased from APExBIO Technology LLC (Houston, USA). Streptozotocin (STZ) was purchased from Sigma‒Aldrich (USA). Antibodies against Nrf2, p-Nrf2, β-actin, heme oxygenase 1 (HO-1), and GPX4 were from Proteintech Group, Inc. (USA). The Iron Assay Kit was purchased from BioAssay Systems (Hayward, CA, USA). ROS, MDA, and GSH assay kits were from Nanjing Jiancheng Biotechnology Co., Ltd. (Nanjing, China).

### Animals

Eighty four C57BL/6J mice (male, 5 weeks) were purchased from Hunan Slake Jingda Laboratory Animal Co., Ltd. (Changsha, China). The animals (4 mice/cage) were kept in an environment with a 12-h light/dark cycle (lights on at 7:00 am) and a relative humidity of 60–65% at 25 ± 1 °C and had free access to food and water. Animal protocols were conducted strictly according to the principles launched by the China Council on Animal Care and approved by the Animal Experimentation Ethics Committee of the University of South China (Permit Number: XYXK201904015).

### T2DM mouse model establishment

T2DM mouse models were induced by using C57BL/6J mice as described in our previous works (Gu et al. 2019). Briefly, the animals were divided into normal control group (n = 12) and T2DM (n = 72) group. Normal control group animals were fed a chow diet, while T2DM group animals were fed a high fat diet (HFD) throughout the experiment. After feeding for 4 weeks (Gu et al. 2019; Liu et al. 2019), T2DM group mice were intraperitoneally (i.p.) injected with a single 50 mg/kg dose of STZ. Normal control mice were injected with an equal volume of sodium citrate buffer (sodium citrate buffer was used as the solvent of STZ) only. Fasting blood glucose levels (6 h fast) before the experiment and 3 days after STZ injection were determined by enzymatic glucose oxidase peroxidase diagnostic kits. Mice with glucose levels above 16.67 mmol/L were considered diabetic.

## Experimental designs

### Experiment 1

In experiment 1, we illustrated whether Art ameliorated cognitive deficits and Nrf2-mediated ferroptosis of hippocampal neurons in T2DM mice. STZ treatment two weeks later, the mice exhibited T2DM. These T2DM mice were further randomly divided into T2DM group and T2DM + Art group (n = 12 per group). The mice were given Art (40 mg/kg, i.p.) or an equal volume of vehicle (5% DMSO solution) once daily for 4 consecutive weeks. The selected dosage of Art was based on previous studies (Lin et al. 2021; Qiang et al. 2018). After the Y-maze test (YMT) Morris water maze test (MWMT), hippocampal tissues were harvested for hematoxylin and eosin (H&E) staining, transmission electron microscopy (TEM) analysis, and measurement of the iron, ROS, MDA and GSH concentrations by kits. The protein levels of p-Nrf2 and GPX4 were detected by western blotting.

### Experiment 2

In this experiment, we confirmed whether Art reversed T2DM-induced cognitive deficits by inhibiting Nrf2-mediated ferroptosis of hippocampal neurons. The T2DM mouse models were established as described in experiment 1. At the end of week 6, mice (n = 12 per group) were injected with vehicle (5% DMSO solution, i.p.), Art (40 mg/kg, i.p., once daily), or ML385 (30 mg/kg, i.p., once every other day). and erastin (30 mg/kg, i.p., once every other day) for 4 consecutive weeks. The selected dosage of ML385 and erastin, and the rationale behind their decision to administer treatment for four weeks were based on efficacy in murine studies (Wei et al. 2023; Yan et al. 2022). The mice were then subjected to YMT and MWMT, followed by H&E staining, TEM observation, iron assay, oxidative stress relative tests, and Western blot analysis as described in experiment 1.

### Behavioral tests

#### Y-maze test

The YMT was performed to evaluate hippocampus-dependent spatial memory as described in previous studies (Guan et al. 2017; Maki et al. 2018). The apparatus (45 cm × 14 cm × 15 cm) is a chamber with A, B, and C arms (at a 120° angle to each other). During the adaptation period, each mouse was allowed to explore the apparatus for 10 min. During the subsequent test session, each mouse was gently placed in the central point of the maze and permitted to freely explore for 5 min. The behavioral test data were recorded by a video camera and analysed by the software SuperMaze (Shanghai, China). Spatial memory performance was measured by the index of spontaneous alternation. Percentage of alternation (%) = number of sequential triplet entrances/total entries × 100 (Zou et al. 2017).

#### Morris water maze test

MWMT was performed to measure learning and memory function as previously described (Gu et al. 2019). The experimental device is composed of a cylindrical pool that is artificially divided into four quadrants. On the 1st day, each mouse was trained to locate the visible platform. From day 1 to day 4, each mouse was placed in one of the four quadrants of the pool to find the hidden platform within 120 s. If the mice failed to locate the hidden platform, they were guided to it and remained there for 30 s. On the 5th day, memory retention was assessed by a probe trial, during which the hidden platform was removed, and each mouse was allowed to find the target quadrant for 90 s. After the probe trials, a visible platform test was performed to evaluate visual and sensorimotor functions. The swimming routes, escape latency to find the platform, and total time in the targeted quadrant were recorded by a video-assisted tracking system. The data were analysed by using the MT-200 Morris image motion system (Chengdu, China).

#### H&E staining

After anaesthetizing by inhalation of isoflurane, the mice were transcardially perfused with phosphate-buffered saline (PBS) and then perfused with 4% paraformaldehyde. Whole brain tissues were isolated, followed by fixation and dehydration. Subsequently, the dehydrated samples were embedded in paraffin and sectioned coronally at 4 µm. The sections were then subjected to hematoxylin and eosin (H&E) staining to visualize the hippocampal tissue structure. The changes in pyramidal neuron morphology and neuron number in the hippocampal cornu ammonis 1 (CA1) region were analysed under a light microscope (200 magnification). The number of pyramidal neurons in the CA1 area was measured as we previously described (Gu et al. 2019).

#### TEM observation

The ultrastructure in the hippocampus was observed as we described previously (Gu et al. 2019). The isolated hippocampal CA1 tissue was cut into 1 mm^3^ blocks. Briefly, the blocks were fixed with 2.5% glutaraldehyde for 6 h, followed by postfixation with 1% osmium tetroxide for 1 h. After fixation, rinsing, dehydration, and embedding, the samples were cut into 50 nm ultrathin sections. Subsequently, the sections were stained with 3% uranyl acetate and lead citrate. For each sample, 8 sections were observed by a transmission electron microscope (TEM, JEM-1200EX; JEOL, Tokyo, Japan).

#### Measurement of iron, ROS, MDA and GSH levels by kits

An appropriate amount of hippocampal tissue was ground by a vibrating homogenizer with ice cold buffer. The grinding solution was centrifuged for 10 min, and then the supernatant was collected to determine the iron (Fe^2+^), ROS, MDA and GSH concentrations according to the manufacturer's instructions for the test kits. The optical density of iron, ROS, MDA, and GSH was determined at 570 nm, 500 nm, 532 nm, and 405 nm by a Thermo Scientific Multiskan FC microplate reader (Shanghai, China). The concentrations of iron, ROS, MDA, and GSH were normalized to the weight of tissue samples.

#### Western blot analysis

Western blot analysi**s** was performed to determine the protein expression of Nrf2, HO-1, and GPX4 in the hippocampus of the mice. The collected hippocampal tissues were lysed in RIPA lysis buffer with phosphatase and protease inhibitors, followed by centrifugation to collect the supernatant. Total protein content was quantified by a BCA protein assay kit. A total of 20 µg of protein was subjected to sodium dodecyl sulfate‒polyacrylamide gel electrophoresis. The separated proteins were transferred to polyvinylidene difluoride membranes and then blocked with 5% skim milk for 1 h. Subsequently, the membranes were incubated with primary antibodies against Nrf2 (1:1000), HO-1 (1:1000), GPX4 (1:1000), and β-actin (1:1000) overnight at 4 ℃. After washing three times with TBST, the blots were incubated with horseradish peroxidase (HRP)-labelled secondary antibodies against rabbit/mouse for 1 h. The bands were detected by an enhanced chemiluminescence detection system. The band density was quantified by Bio-Rad software and normalized to the β-actin level.

#### Statistical analysis

All data are presented as the mean ± SEM and were analysed by GraphPad Prism 9 software. For the behavioral data, statistical analyses were performed using two-way analysis of variance (ANOVA) followed by a Bonferroni post hoc test. Additional data in this study were analysed by Student’s t test to compare two groups or one-way ANOVA to compare multiple groups followed by the least significant difference (LSD) post hoc test. Statistical significance was set at *P* < 0.05.

## Results

### Art alleviates cognitive impairment and hippocampal neuronal loss in T2DM mice

**Fig. 1:**
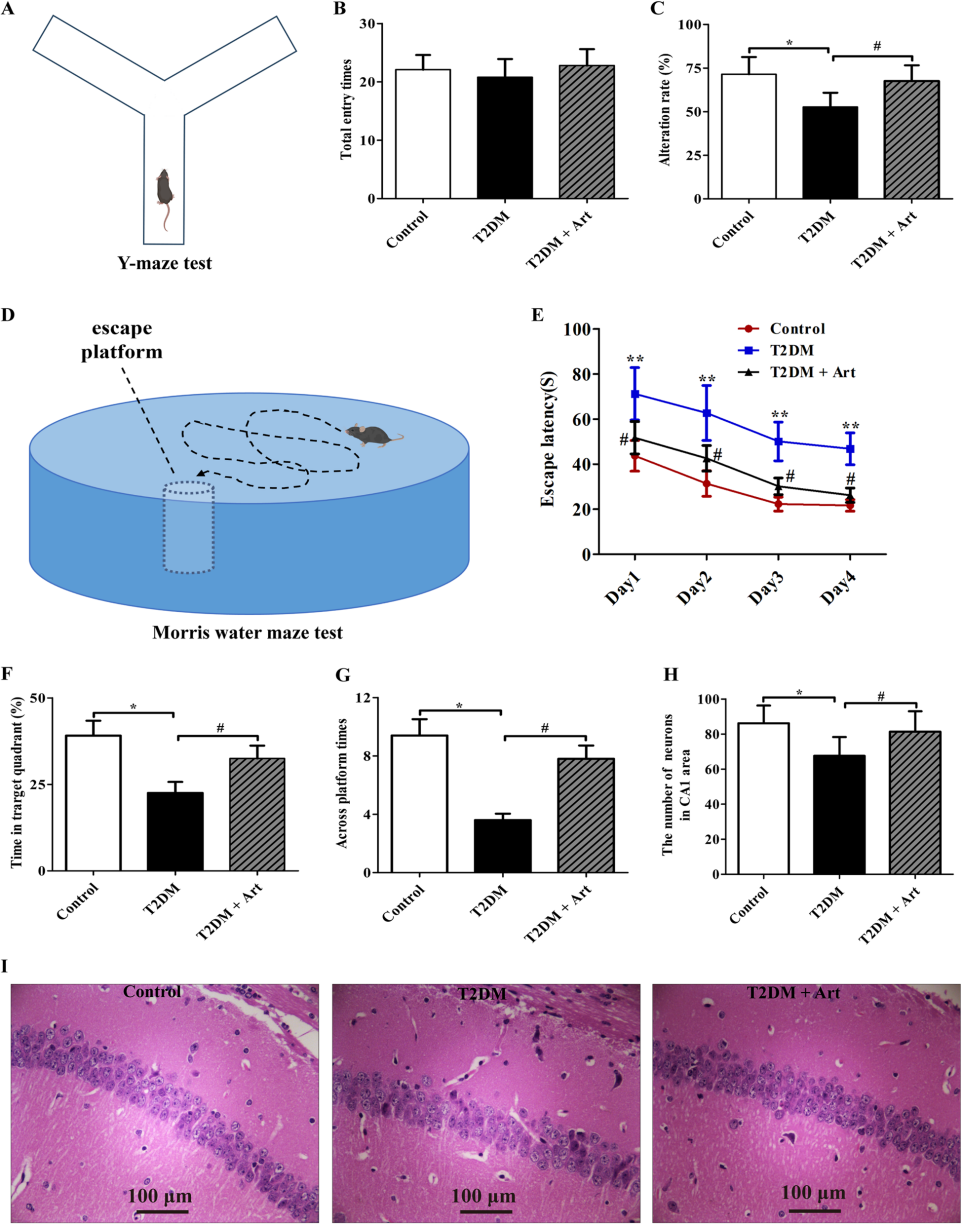
Artemisinin improves cognitive function and attenuates hippocampal neuronal loss in T2DM mice. T2DM mice were treated with artemisinin (Art, 40 mg/kg/d, i.p.) for 4 weeks. **A** Schematic diagram of Y-maze test. **B** The total number of times the mice entered the arms and (**C**) the alternation rate were analyzed in the Y maze test. **D** Schematic diagram of Morris water maze test. **E** The escape latency to find the platform in the navigation phase, **F** time spent in the target quadrant, and (**G**) the number of platform crossings during the probe trial phase in the MWM test were also analyzed. Data are expressed as the mean ± SEM (n = 12 per group). *P < 0.05, **P < 0.01 vs the control group; ^*#*^*P* < 0.05 vs the T2DM group. **H** Statistical analyses for neuron number in the CA1 region. **I** Representative H&E staining images from the field CA1 region of the mice. Scale bar, 100 μm. The data are expressed as the mean ± SEM (n = 4 per group). *P < 0.05

We first explored the beneficial role of Art in the cognitive performance of T2DM mice by YMT (Fig. 1A). As shown in (Fig. 1B), there was no significant difference in the number of total entries among the control group, T2DM group, and T2DM + Art group. However, T2DM mice presented a notable decrease in the alternation ratio compared with the control mice (Fig. 1C). As expected, T2DM mice treated with Art exhibited a higher alternation rate than those treated with vehicle. In the MWMT (Fig. 1D), we demonstrated that the escape latency of each group to find the hidden platform was shortened over a 4-day training period (Fig. 1E). However, the swimming routes were obviously complex, and the escape latency to reach the platform was notably prolonged in the T2DM group compared with the control group. Additionally, compared with the normal control group, the average time spent in the target quadrant and the number of times crossing the platform in the T2DM group were significantly reduced (Fig. 1F, G). Interestingly, Art treatment significantly shortened the escape latency to the platform and increased the number of platform crossings. These results show that Art can attenuate cognitive defects in T2DM mice.

Hippocampal neuronal loss, especially in the CA1 region, is closely associated with diabetic cognitive dysfunction (Hansen et al. 2015). To further confirm the neuroprotective effects of Art on T2DM mice, neuronal cell injury and loss were assessed by H&E staining. Compared with the mice in the normal control group, neuronal cell damage and loss in T2DM mice were significantly exacerbated, as indicated by the irregular arrangement of neurons and the decrease in the number of neurons (Fig. 1H, I). However, treatment with Art reversed these pathomorphological changes in the hippocampal CA1 region. These results reveal that Art protects neurons in the CA1 area from T2DM-induced damage.

### Art ameliorates oxidative stress and neuronal ferroptosis in the hippocampal CA1 region of T2DM mice

**Fig. 2:**
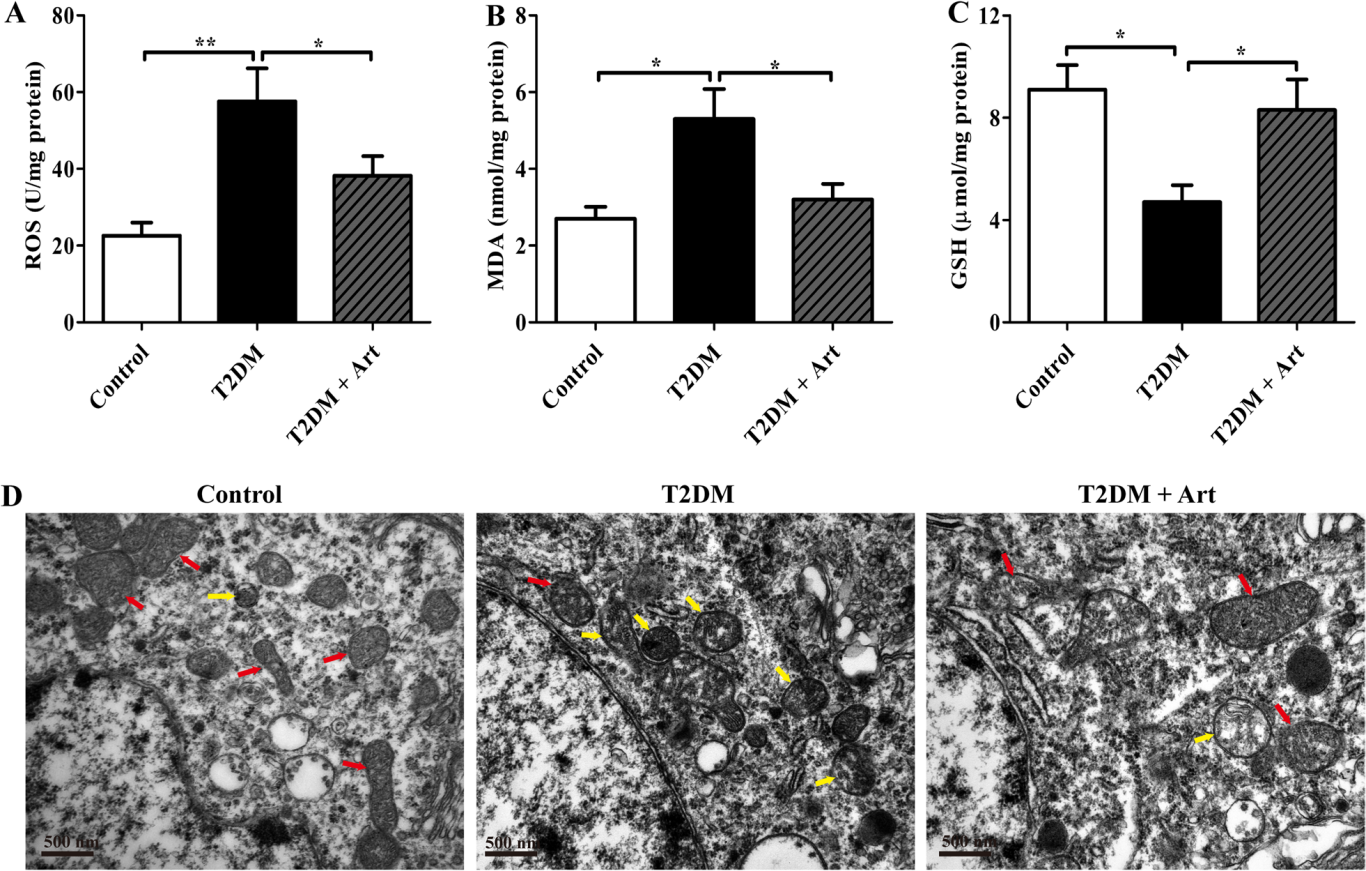
Artemisinin attenuates oxidative stress and ferroptosis in the hippocampal CA1 region of T2DM mice. T2DM mice were treated with artemisinin (Art, 40 mg/kg/d, i.p.) for 4 consecutive weeks. **A–C** ROS, MDA, and GSH concentrations in the hippocampal CA1 area were detected using assay kits. **D** Representative transmission electron microscopy (TEM) images of mitochondrial morphology (normal mitochondria are indicated with red arrows, and shrunken and broken mitochondria are indicated with yellow arrows) in the hippocampal CA1 area of the mice. Scale bar, 500 nm. The data are expressed as the mean ± SEM (n = 4 per group). *P < 0.05, **P < 0.001

Oxidative stress is one of the major factors contributing to T2DM-induced neuronal injury and loss. Ferroptosis, a novel form of cell death, is characterized by iron-dependent lipid peroxidation. To reveal the mechanism underlying the neuroprotective effects of Art on T2DM mice, the levels of oxidation products (ROS and MDA) and the antioxidant GSH in the hippocampus were measured by biochemical detection, and neuronal ferroptosis in the hippocampal CA1 region was observed by TEM. As shown in Fig. 2A–C, compared with those in the control group, ROS and MDA levels in the hippocampus of the T2DM group were significantly increased, but the GSH concentration was dramatically decreased. Furthermore, our TEM results illustrated that neurons in the hippocampal CA1 region of the T2DM group displayed smaller, shrunken and broken mitochondria (Fig. 2D), which is a critical morphological feature of ferroptosis. Interestingly, Art treatment profoundly improved the neuronal morphological characteristics of mitochondria. Collectively, these results demonstrate that Art notably prevents T2DM-induced neuronal ferroptosis in the hippocampal CA1 region.

### Art upregulates the protein expression of Nrf2, HO-1, and GPX4 in the hippocampus of T2DM mice

**Fig. 3:**
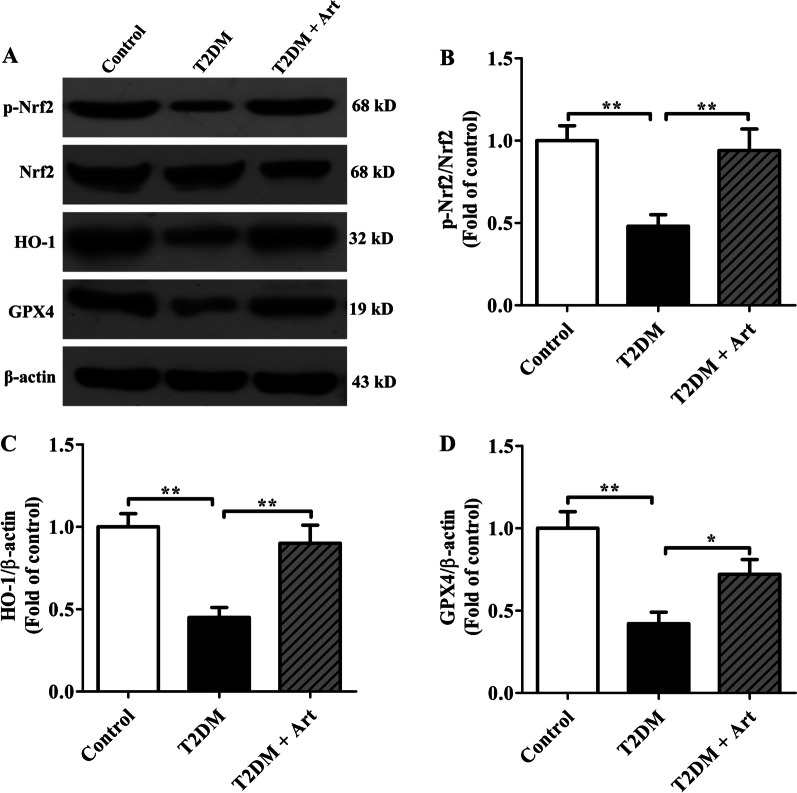
Artemisinin increases the protein levels of Nrf2, HO-1, and GPX4 in the hippocampal CA1. T2DM mice were treated with artemisinin (Art, 40 mg/kg/d, i.p.) for 4 consecutive weeks. **A** Western blot analysis was performed to measure the expression of p-Nrf2, Nrf2, HO-1, and GPX4. **B**–**D** The relative optical density values of Nrf2, HO-1 and GPX4 proteins were quantified by NIH ImageJ software. β-actin was used as a control for protein loading. fold of control: relative fold change compared to the control group. The data are expressed as the mean ± SEM (n = 4 per group). *P < 0.05, **P < 0.001

The Nrf2/HO-1/GPX4 pathway plays a crucial role in preventing oxidative stress in cells by inhibiting the production of intracellular ROS, and we confirm that Art can attenuate the oxidation state in the hippocampus of T2DM mice. Hence, the influence of Art on the expression of this signaling pathway was determined by Western blotting analysis. Our results showed that p-Nrf2 protein levels were significantly decreased in the T2DM group compared with the control group (Fig. 3A, B). Consistent with the decrease in p-Nfr2 protein levels, the protein expression levels of HO-1 and GPX4 were also lower in the T2DM group than in the control group (Fig. 3C, D). As expected, the protein level of the activated form of the transcription factor, p-Nrf2, was markedly elevated after Art treatment for 4 weeks, but the levels of total Nrf2 protein did not increase significantly compared to the T2DM group. Moreover, Art treatment notably enhanced the expression of HO-1 and GPX4 in the hippocampus of T2DM mice. These results demonstrate that Art upregulates the expression of HO-1 and GPX4 by promoting Nrf2 nuclear translocation, thereby preventing oxidative stress in the hippocampus of T2DM mice.

### ML385 or erastin can abolish the inhibitory effect of art on hippocampal neuronal ferroptosis in T2DM mice

**Fig. 4:**
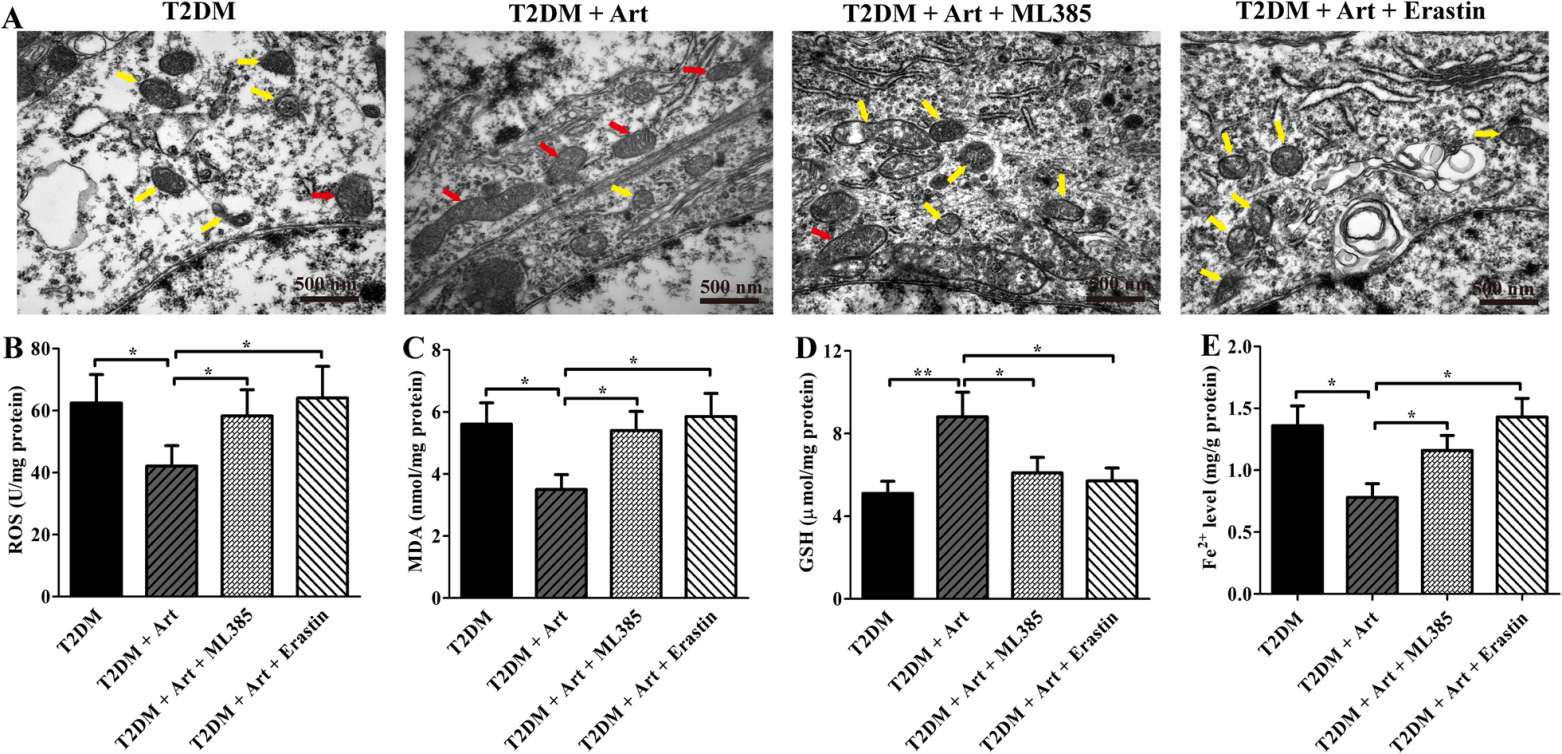
ML385 or erastin treatment abolishes the inhibitory effect of Art on neuronal ferroptosis in the hippocampus. T2DM mice were coadministered artemisinin (Art, 40 mg/kg/d, i.p.) and ML385 (30 mg/kg, i.p.) or erastin (30 mg/kg, i.p.) for 4 consecutive weeks. **A** Representative transmission electron microscopy (TEM) images of mitochondrial morphology (normal mitochondria are indicated with red arrows, and shrunken and broken mitochondria are indicated with yellow arrows) in the hippocampal CA1 area of the mice. Scale bar, 500 nm. **B**–**E** ROS, MDA, GSH, and Fe^2+^ contents in the hippocampal CA1 area were detected using assay kits. The data are expressed as the mean ± SEM (n = 4 per group). *P < 0.05, **P < 0.001

To further illustrate the neuroprotective role of Art by activating Nrf2 to inhibit neuronal ferroptosis, T2DM mice were treated with the Nrf2 inhibitor ML385 or ferroptosis inducer erastin. Morphological changes in the mitochondria of neurons in the hippocampal CA1 area were observed, and lipid peroxide levels and iron content in the hippocampus were measured. As shown in Fig. 4A, after cotreatment with Art and ML385, neuronal mitochondria in the CA1 region of T2DM mice were smaller than those in diabetic mice treated with Art alone. Additionally, Art and ML385 cotreated T2DM mice exhibited serious lipid peroxidation, poor antioxidant capacity, and high iron content compared with the mice treated with Art alone (Fig. 4B–E). Similar trends were observed in Art and erastin cotreated T2DM mice. Taken together, these results indicate that Art can activate Nfr2 to inhibit neuronal cell ferroptosis in the hippocampal CA1 region.

### ML385 or erastin reverses the ameliorative effect of Art on T2DM-induced neuronal loss in the hippocampal CA1 region

**Fig. 5:**
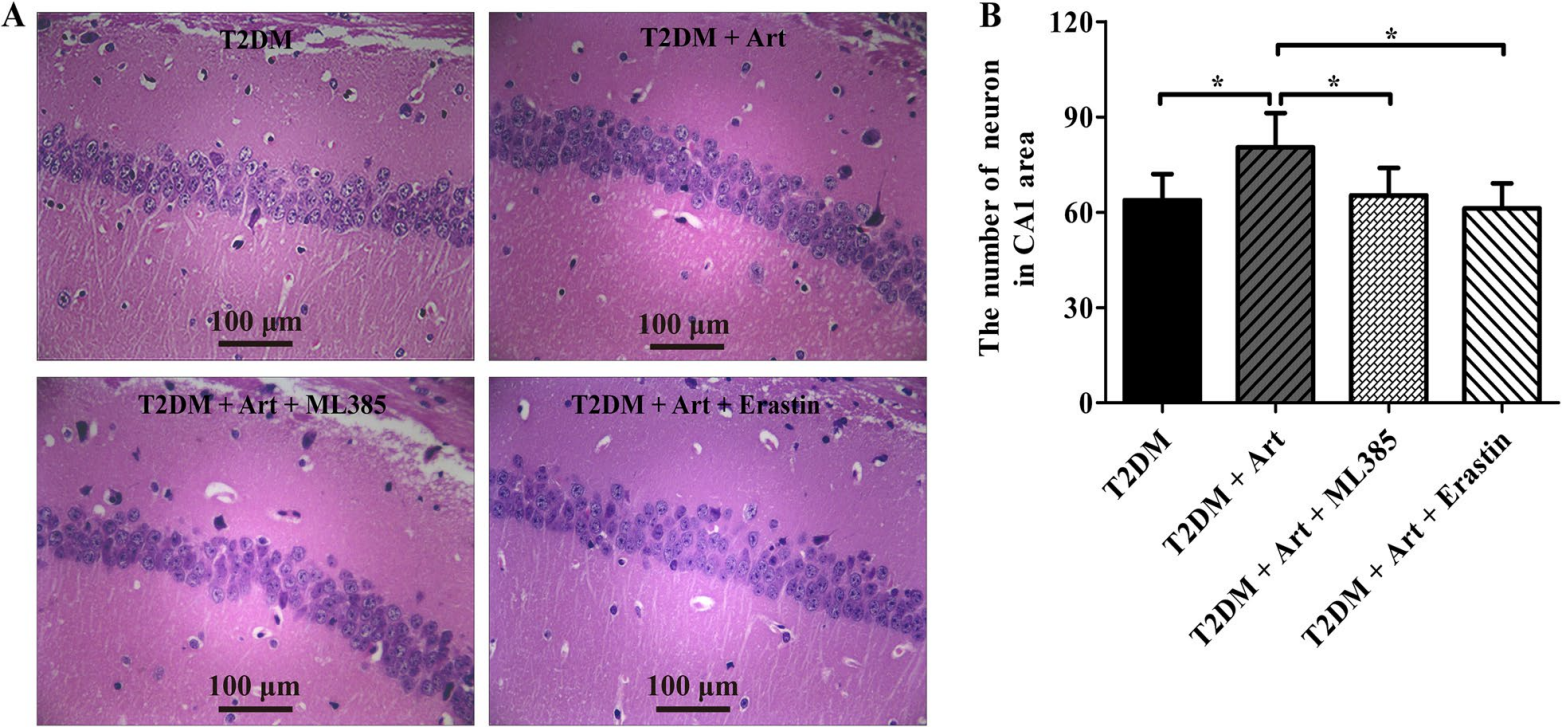
Cotreatment with ML385 or erastin abolishes the neuro-protective effect of Art on T2DM mice. T2DM mice were cotreated with artemisinin (Art, 40 mg/kg/d, i.p.) and ML385 (30 mg/kg, i.p.) or erastin (30 mg/kg, i.p.) for 4 consecutive weeks. Neuronal injury and neuron loss in the hippocampus were measured by H&E staining. **A** Representative images of the hippocampal CA1 region by H&E staining. Scale bar, 100 µm. **B** Statistical analyses for neuron number in the CA1 region. The data are expressed as the mean ± SEM (n = 4 per group). *P < 0.05

To confirm whether Art prevented neuronal cell loss by activating Nrf2 to inhibit ferroptosis, H&E staining was used to evaluate neuronal cell damage and death in the hippocampal CA1 region of T2DM mice after coadministration of Art with the Nrf2 inhibitor ML385 or ferroptosis inducer erastin. As shown in Fig. 5, the morphological features of neurons were improved, and the number of neurons was significantly increased in the Art treatment group compared with the T2DM group. However, the protective effect of Art on hippocampal neurons was abolished by cotreatment with ML385 or erastin. Hence, these results reveal that activating Nrf2 to inhibit ferroptosis is a major mechanism by which Art ameliorates T2DM-induced neuronal cell loss in the CA1 area.

### ML385 or erastin blocks the ameliorative effect of Art on cognitive deficits in T2DM mice

**Fig. 6:**
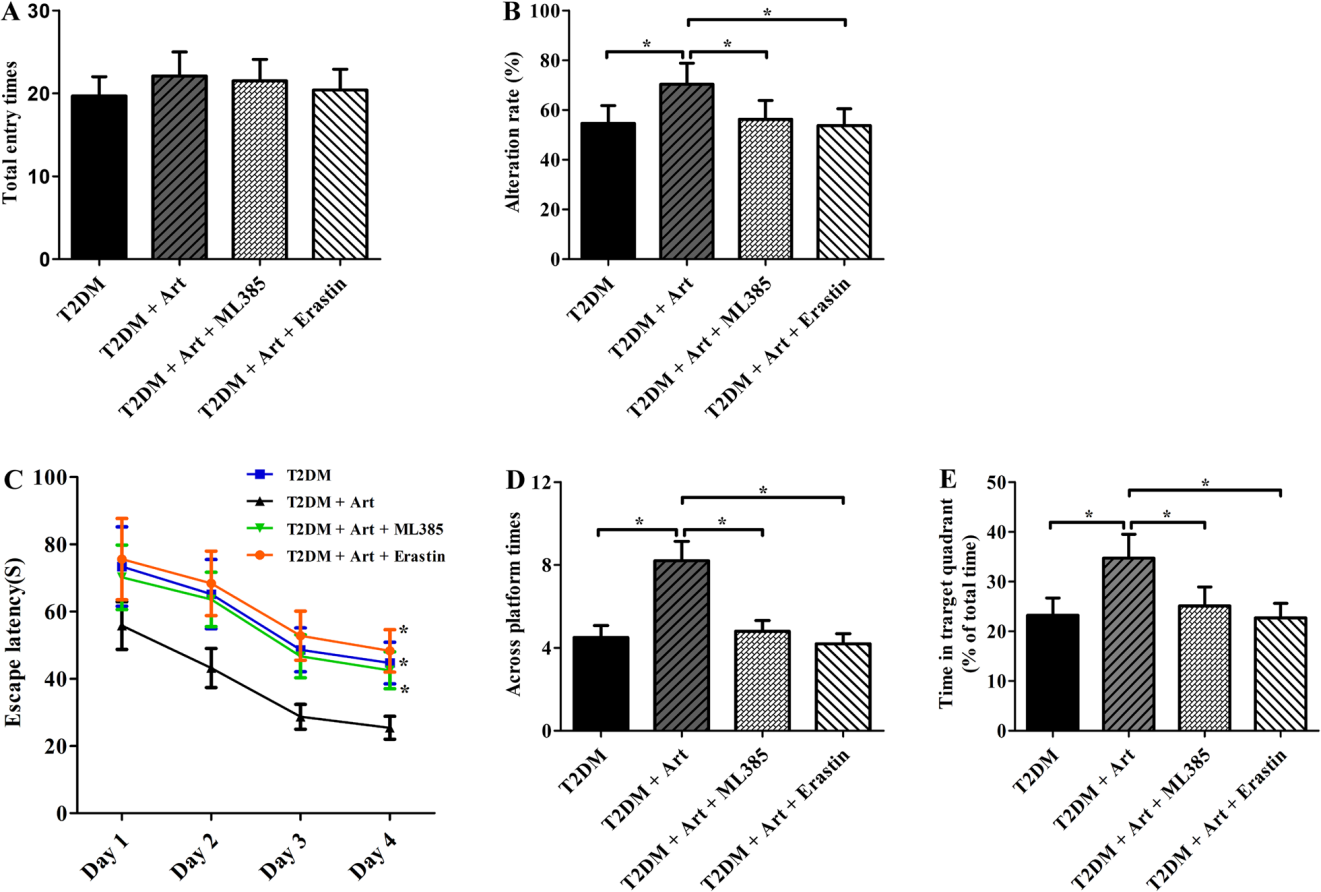
Cotreatment with ML385 or erastin reverses the ameliorative effect of Art on cognitive deficits in T2DM mice. T2DM mice were cotreated with artemisinin (Art, 40 mg/kg/d, i.p.) and ML385 (30 mg/kg, i.p.) or erastin (30 mg/kg, i.p.) for 4 consecutive weeks. **A** Total entry times and (**B**) the alternation rate were detected in the Y maze test. **C** The escape latency to find the platform in the navigation phase, **D** time spent in the target quadrant and (**E**) number of platform crossings during the probe trial phase in the MWM test were also analysed. Data are the mean ± SEM (n = 12 per group). *P < 0.05 vs the T2DM + Art group

Finally, we determined whether ML385 or erastin can abolish the protective role of Art in T2DM-induced cognitive impairment using the YMT and MWMT. As shown in Fig. 6B, the alteration rate of the T2DM + Art group was notably increased when compared with that of the T2DM group. In the MWMT, the escape latency in the ML385 and erastin groups was significantly prolonged (Fig. 6C), but the number of platform crossings and swimming time in the target quadrant were dramatically reduced compared with those of the mice in the Art treatment alone group (Fig. 6D, E). Taken together, these results show that both ML385 and erastin can block the ameliorative effect of Art on learning and memory deficits in T2DM mice.

## Discussion

**Fig. 7:**
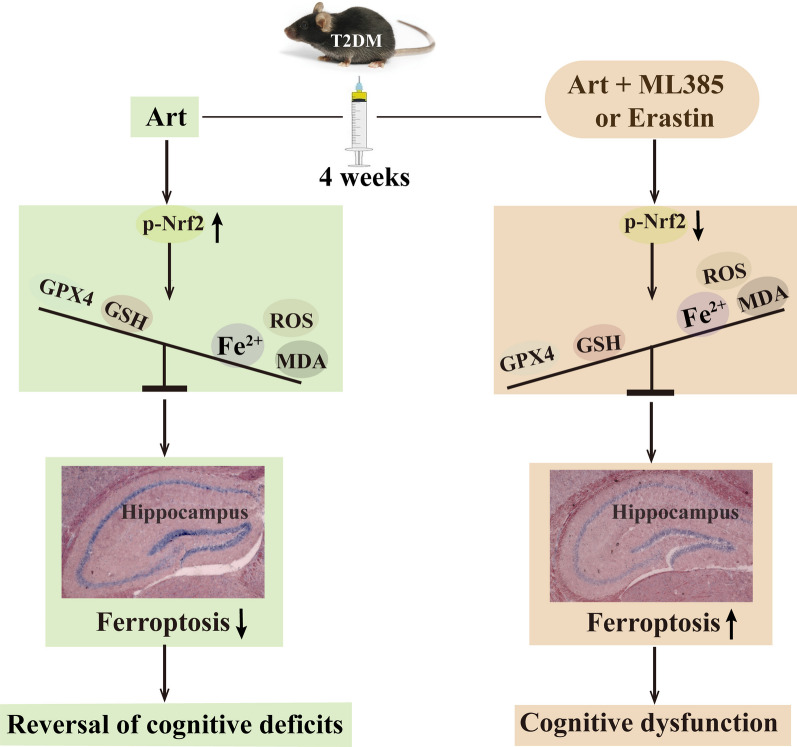
Proposed mechanisms of Artemisinin (Art) on anti-ferroptosis in hippocampal neurons of T2DM mice. Artemisinin (Art) promotes the activation of Nrf2 to increase GPX4/GSH expression and inhibits lipid peroxidation and iron overload in the hippocampus, thereby suppressing hippocampal neuronal ferroptosis, which contributes to the reversal of cognitive deficits in T2DM mice

In the present work, we explored the effect of Art on T2DM-induced cognitive deficits in mice and the underlying mechanism. The results indicated that Art significantly improved the learning and memory performance of T2DM mice, ameliorated neuronal cell loss, oxidative stress and neuronal ferroptosis, and increased the protein expression of Nrf2, HO-1 and GPX4 in the hippocampus. Furthermore, administration of the Nrf2-specific inhibitor ML385 or the ferroptosis-specific inducer erastin not only reversed the ameliorative effects of Art on T2DM-induced hippocampal neuronal cell loss, oxidative stress, and ferroptosis but also abolished the protective effects of this agent on the cognitive functions of the mice. Taken together, these results reveal that the neuroprotective role of Art in diabetic cognitive impairments is associated with the inhibition of hippocampal neuron ferroptosis via Nrf2 activation (Fig. 7).

The pathological injury and loss of neurons in the hippocampus, especially in the CA1 region, play a critical role in diabetes-associated cognitive impairment (da Costa et al. 2013; Hansen et al. 2015; Sima and Li 2005). Due to the protective effects of Art on hippocampal neurons, this agent in ameliorating diabetic cognitive deficits has gradually received attention (Poorgholam et al. 2023). Hence, in this work, we first clarified the effects of Art on cognitive decline and hippocampal neuronal loss in T2DM mice. Our MWMT results indicated that Art treatment significantly reduced the escape latency of diabetic mice during the training trial and increased the number of crossing platform times of diabetic mice in the space probe trial. Furthermore, the results of H&E staining illustrated that diabetic mice obviously exhibited neuronal damage and loss in hippocampal CA1 regions, and those hippocampal pathological changes were significantly reversed by Art treatment. These results reveal that Art improves cognitive performance and attenuates hippocampal neuronal damage in T2DM mice. Previous studies have demonstrated that Art can improve neuronal functions and cognitive performances of Alzheimer's disease in mouse model (Zhao et al. 2022; Zhao et al. 2020). The present study reveals for the first time that Art profoundly prevents hippocampal neuronal death and cognitive decline in T2DM mice.

Ferroptosis, a novel form of cell death, is characterized by iron-dependent lipid peroxidation, which triggers oxidative stress and subsequent neuronal damage and loss (Duan et al. 2022; Yang et al. 2021). Mechanisms responsible for ferroptosis include the inactivity of GPX4 and the accumulation of lipid peroxides (Bersuker et al. 2019; Yang and Stockwell 2016; Zou et al. 2020). Indeed, these critical biochemical characteristics of ferroptosis are present in diabetic brain tissues, especially in the hippocampus (Xie et al. 2023). Therefore, monitoring intracellular iron and ROS levels and the expression of GSH and GPX4 are the major approaches to measure ferroptosis. In addition, observation of changes in mitochondrial morphology by TEM is also regarded as an accessible and accurate way to evaluate ferroptosis (Yang et al. 2021). Studies have indicated that decreases in the antioxidant function of GSH and GPX4 are critical contributors to ferroptosis (Bersuker et al. 2019; Lai et al. 2022; Yang and Stockwell 2016). Given that enhancing GPX4 expression can prevent ferroptosis and ameliorate hippocampal neuron loss and cognitive impairment, targeting ferroptosis may be a valid therapeutic strategy for neurodegenerative diseases. Indeed, increasing evidence has shown that ferroptosis inhibitors such as iron chelating agents, and antioxidants can prevent the development of neurodegenerative diseases (Hao et al. 2021; Wang et al. 2023).

To clarify whether ferroptosis in the hippocampus of diabetic mice occurred in the hippocampus of diabetic mice, we analysed iron content, ROS, MDA and GSH levels, and GPX4 expression. The results indicated that ferroptosis was implicated in diabetes-induced hippocampal neuronal damage and loss, as evidenced by the increases in iron concentrations and MDA and ROS contents, together with the reduction in GSH and GPX4 levels. These indicators suggest that neuronal ferroptosis may occur in the hippocampus of diabetic mice. Moreover, this hypothesis was further supported by our TEM results, which demonstrated that mitochondria in the hippocampus of T2DM mice were shrunken and lacked ridges. These mitochondrial features were in agreement with those previously reported for ferroptosis (Yang et al. 2021). Taken together, these findings reveal that neuronal ferroptosis occurs in the hippocampus of T2DM mice.

The results of this work indicated that Art improved the cognitive function of T2DM mice by activating Nrf2 to mitigate hippocampal neuronal ferroptosis. Several recent studies have shown that Art can protect cells from oxidative stress-induced damage by inhibiting ROS production and promoting antioxidant enzyme activity (Cao et al. 2020; Hua et al. 2022; Yan et al. 2021). Moreover, this agent and its derivatives have been identified as potential ferroptosis regulators to treat hepatic fibrosis and tumors (Kong et al. 2019). However, whether Art can prevent hippocampal neuronal ferroptosis and ameliorate cognitive deficits in T2DM mice deserves to be studied. As expected, Art treatment significantly attenuated diabetes-induced hippocampal neuronal loss and cognitive impairment in mice. Of note, treatment with this agent obviously ameliorated hippocampal neuronal ferroptosis, as evidenced by decreases in hippocampal iron concentration and MDA and ROS levels and increases in GSH and GPX4 expression. Furthermore, TEM results confirmed that the morphology of mitochondria in the hippocampal neurons of diabetic mice was improved after Art treatment. However, these neuronal protective effects of Art on neuron survival and cognitive performance of T2DM mice were reversed by the ferroptosis inducer erastin.

Finally, we further explored whether the anti-ferroptosis effect of Art in T2DM mice occurs by activating Nrf2, which plays a crucial role in preventing cells from oxidative damage and ferroptosis. Given that the activity of Nrf2 is not enough to antagonize oxidative stress, it often contributes to various pathological injuries. Indeed, emerging evidence has shown that relative Nrf2 deficiency is involved in the pathogenesis of diabetes and its complications (Chang et al. 2012; Wu et al. 2020). In the present work, we demonstrated that inactivation of Nrf2 signaling indeed occurred in the diabetic hippocampus, as evidenced by a reduction in nuclear Nrf2 protein levels, HO1 and GPX4 expression. Wang et al. reported that the activity of Nrf2 significantly declined in the hippocampus of diabetic mice (Wang et al. 2021). Therefore, these data suggest that insufficient activity of Nrf2 results in the accumulation of ROS and MDA, thereby promoting hippocampal neuronal ferroptosis and exacerbating cognitive impairment. As expected, Art treatment significantly increased the expression of Nrf2 and the downstream antioxidants HO1 and GPX4. However, these anti-oxidative and -ferroptotic effects of Art were abrogated by coadministration of the Nrf2-specific inhibitor ML385.

Although our work provided new insights into the mechanism by which Art ameliorated cognitive decline in T2DM mice, it has some limitations. First, we only performed studies on male mice, but not on female mice in the present work. This is due to the difficulty in the hormonal control of female mice. Actually, the gender differences in T2DM-associated cognitive decline do exist (Chatterjee et al. 2016). Second, we did not explore the neuro-protective effect of intracerebroventricular injection of Art on TDM2 mice. These experiments will be further performed in a follow-up study.

## Conclusions

This study indicated that neuronal ferroptosis may be implicated in the pathogenesis of T2DM-induced cognitive deficits. Notably, our results demonstrated that Art improved diabetic cognitive functions by inhibiting ferroptosis. Furthermore, we found that Art attenuated diabetes-associated neuronal ferroptosis by activating the Nrf2 pathway. Consequently, our data highlight a beneficial role of Art in diabetic cognitive impairment and provide a promising strategy for preventing and treating T2DM-induced cognitive dysfunction via the regulation of neuronal ferroptosis.

## Data Availability

The datasets used and/or analysed during the current study are available from the corresponding author on reasonable request.

## Abbreviations

Art: Artemisinin
DMSO: Dimethyl sulfoxide
GPX4: Glutathione peroxidase 4
GSH: Glutathione
H&E: Hematoxylin and eosin
HO-1: Hemeoxygenase 1
HRP: Horseradish peroxidase
i.p.: Intraperitoneally
MDA: Malondialdehyde
MWMT: Morris water maze test
Nrf2: Nuclear factor erythroid-2-related factor 2
ROS: Reactive oxygen species
STZ: Streptozotocin
T2DM: Type 2 diabetes mellitus
YMT: Y-maze test
